# Characterization of Delirium and Its Severity Across Three Distinct Clinical Settings

**DOI:** 10.1093/geroni/igaf122.3895

**Published:** 2025-12-31

**Authors:** Jason Albaum, Tamara Fong, Richard Jones, Sharon Inouye

**Affiliations:** Marcus Institute for Aging Research, Hebrew SeniorLife, Boston, Massachusetts, United States; Beth Israel Deaconess Medical Center, Harvard Medical School, Boston, Massachusetts, United States; Brown University, Brown University, Rhode Island, United States; Marcus Institute for Aging Research, Hebrew SeniorLife, Harvard Medical School, Boston, Massachusetts, United States

## Abstract

Delirium and its severity (i.e., the intensity or degree of delirium symptoms) can be difficult to measure in people with dementia due to substantial overlap in symptoms and clinical features, complicating both diagnosis and research. In this descriptive study, we used data from the Better Assessment of Illness II (BASIL II) study of adults aged ≥70 years recruited from medical inpatient (Site 1), inpatient elective surgery (Site 2), or skilled nursing facility (Site 3) settings, to characterize delirium and its severity in settings with varying dementia prevalence. Trained staff conducted patient and proxy interviews, chart review, and delirium assessments at baseline and 1-2 days after acute illness or surgery using the Confusion Assessment Method (CAM; delirium present/absent) and CAM-Severity (CAM-S; scored 0-19, 19 = worst). Among 488 participants (mean [SD] age 79 [6] years; 58% female; 75% white), dementia prevalence was 23% (Site 1), 11% (Site 2), and 83% (Site 3). Following acute illness or surgery, the proportion meeting criteria for delirium and median (IQR) CAM-S scores among those with delirium were: Site 1, 50%, score 6 (4-6); Site 2, 1%, score 3 (3-3); Site 3, 24%, score 8 (6-10). Delirium rates and severity showed different patterns of variation across settings. Dementia prevalence may be associated with greater delirium severity; however, it is possible that chronic cognitive symptoms may also elevate delirium severity scores. These findings underscore the pressing need for studies validating measures of delirium severity in populations with dementia to advance research and patient care.

